# Role of Gonadotropin-Releasing Hormone (GnRH) in Ovarian Cancer

**DOI:** 10.3390/cells10020437

**Published:** 2021-02-18

**Authors:** Carsten Gründker, Günter Emons

**Affiliations:** Department of Gynecology and Obstetrics, University Medicine Göttingen, 37075 Göttingen, Germany; emons@med.uni-goettingen.de

**Keywords:** ovarian cancer, gonadotropin-releasing hormone, GnRH, LHRH

## Abstract

The hypothalamus–pituitary–gonadal (HPG) axis is the endocrine regulation system that controls the woman’s cycle. The gonadotropin-releasing hormone (GnRH) plays the central role. In addition to the gonadotrophic cells of the pituitary, GnRH receptors are expressed in other reproductive organs, such as the ovary and in tumors originating from the ovary. In ovarian cancer, GnRH is involved in the regulation of proliferation and metastasis. The effects on ovarian tumors can be indirect or direct. GnRH acts indirectly via the HPG axis and directly via GnRH receptors on the surface of ovarian cancer cells. In this systematic review, we will give an overview of the role of GnRH in ovarian cancer development, progression and therapy.

## 1. Introduction

Ovarian carcinomas are the eighth most common malignant tumor disease in women diagnosed in the world [1]. Although the risk of developing ovarian cancer in course of their life is only 1.3% for every woman [2], ovarian cancer is the fifth most common cancer-associated cause of death in women [3]. With a 5-year survival rate of 43%, ovarian cancer is the deadliest gynecological disease. Due to the lack of methods for early detection, the disease is diagnosed at an advanced stage in 70% of cases. Only 20% are discovered in stage I and are associated with 5-year survival rates of 90% [4]. Another reason for the late diagnosis is the unspecific symptoms. About 50% of the patients suffer from decreased performance or weight loss [5]. Ovarian cancer is a complex disease that originates from multiple sites [6,7,8]. Besides ovarian surface epithelium, ovarian cancers originate from fallopian tubes [9,10,11,12].

Ovarian cancer is a hormone-dependent disease that is influenced by hormonal signaling pathways [13,14]. This effect asserts itself after the illness and could therefore also have therapeutic potential. Gonadotropin-releasing hormone (GnRH) is pulsatile released from neurons in the hypothalamus and induces expression and pulsatile release of follicle-stimulating hormone (FSH) and luteinizing hormone (LH) from anterior pituitary. Both, FSH and LH in turn promote maturation of follicles, ovulation, corpus luteum formation and synthesis of estrogen and progesterone. In the following, we give an overview on the role of GnRH, the key player in the regulation of ovarian function, in ovarian cancer development and progression. In addition, we shed light on the use of GnRH receptors expressed on the ovarian cancer cell surface as a target of therapy in ovarian cancer.

## 2. Methods

We performed a literature search of PubMed using the terms “GnRH” or “LHRH” or “gonadotropin” and “ovarian cancer”, identifying 381 articles. Abstracts and full papers were screened independently by both authors excluding publications if they were not related to the topic, were double publications or not in English. The remaining 83 publications are described and discussed in this review. Important studies on other tumor entities (42) were included when no such studies on ovarian cancer were available and in addition, its use was necessary for better understanding. Furthermore, publications were discussed which deal with GnRH receptor mechanisms and signaling pathways independent of specific tumor entities (31). Due to the heterogeneity and the limitations of the clinical data, a meta-analysis was not appropriate. Completed and published clinical trials were discussed. A search on clinicaltrials.gov for currently ongoing trials on GnRH and ovarian cancer did not yield any results [15]. Only ongoing studies with other tumor entities were found.

## 3. Results and Discussion

### 3.1. Expression of GnRH and Its Receptor in Ovarian Cancer

GnRH is a peptide consisting of 10 amino acids that plays a main function in the control of human reproduction. The GnRH receptor belongs to class A of the G-protein-coupled receptor family with 7 transmembrane domains [16]. In addition to expression in gonadotropic cells of the pituitary gland and reproductive organs such as myometrium [17] or ovaries [18], expression of GnRH and GnRH receptor could also be detected in some human carcinomas of the urogenital tract. This includes cancers of the ovary [19], endometrium, urinary bladder and prostate [20]. In initial studies, GnRH receptors were found in almost 80% of human epithelial ovarian carcinomas [20,21,22,23]. The serous-papillary ovarian tumor is the most common epithelial tumor disease of the ovary. Histologically, the malignant form is a serous-papillary cystadenocarcinoma. This is divided into the less aggressive low-grade and the more common and more aggressive high-grade subtype. A recent study showed that nearly 90% of patients with high-grade serous ovarian cancer were GnRH receptor-positive [24]. In another study, it was demonstrated that all low-grade (100%) and about 82% high-grade serous ovarian cancer showed GnRH receptor expression [25]. The receptor binding abilities differ between gonadotropic cells in the pituitary and cancer cells. Two types of GnRH coupling sites were found in ovarian cancer cells. The first site has a low affinity and a high capacity. The second binding site has a high affinity with low capacity and is comparable to the GnRH receptor expressed in the pituitary gonadrotrophs [20,26,27]. The low-affinity binding site is similar to the binding site found in the human placenta and corpus luteum. It cannot differentiate between GnRH antagonists and high active GnRH agonists [28]. In addition, the GnRH receptor with low affinity can only be activated with high concentrations of GnRH agonists, while the GnRH receptor with high affinity is already completely activated at low concentrations of GnRH agonists. After the GnRH receptor gene sequence in human pituitary gonadotrophs was shown in 1992 [29], intensive research work was carried out which led to the detection of high-affinity GnRH receptors in ovarian cancer cell lines and in about 80% of primary ovarian cancers [29,30,31,32]. High affinity/low capacity binding sites closely related to the GnRH receptor in the pituitary gonadotrophs have been found in samples of ovarian carcinomas and cell lines expressing mRNA for the GnRH receptor found in the anterior pituitary [30,31,33,34,35,36,37]. Kakar et al. [38] demonstrated that the gene sequence of the GnRH receptor isolated from human ovarian cancers does not differ from that in the pituitary gland. Not only the GnRH receptor was found in these tumors, but also its ligand, GnRH. First of all expression of GnRH mRNA was found in two human breast cancer cell lines [39]. Later, GnRH immunoreactivity, biological activity, and mRNA expression by cell lines and in most tissue samples of ovarian cancers were demonstrated [33,36,37]. As ovarian cancers show expression of GnRH and the GnRH receptor, it seems plausible to speculate about the existence of a paracrine system based on GnRH in many of these cancers. A first very important indication was, on one hand, that treatment with authentic GnRH inhibited the growth of cell lines derived from ovarian cancer. On the other hand, by removing GnRH secreted by the tumor cells by using an anti-GnRH antiserum, proliferation of these cancer cells was upregulated [19].

### 3.2. Direct Effects of GnRH on Ovarian Cancer

Time- and dose-dependent anti-proliferative actions of GnRH agonists have been observed in many cell lines from different cancer entities of the reproductive tract. This includes ovarian cancer [20,21,40,41,42]. GnRH antagonists also show significant growth-inhibiting actions on most GnRH receptor-positive cancer cell lines [20,21]. This indicates that GnRH agonists and antagonists may not be differentiated in the GnRH system in cancer cells. In addition, the signaling mechanisms of the GnRH receptor known to be activated in gonadotropic cells of the pituitary, are not connected in the transmission of the proliferation inhibiting actions of GnRH analogs in cancer cells. In pituitary gonadotrophs, specific binding of GnRH to its receptor leads to a conformational change and subsequent activation of the heterotrimeric G-protein subunit αq/11, which leads to calcium-dependent signal transduction [43,44]. Activation of phospholipase C (PLC) causes the hydrolysis of phosphatidylinositol-4,5-bisphosphate (PIP2). This creates diacylglycerol (DAG) and inositol-1,4,5-trisphosphate (IP3) [45,46,47]. IP3 diffuses from the plasma membrane and activates receptors on the endoplasmic reticulum (ER). This leads to an outflow of calcium from the ER into the cytosol [48,49]. DAG remains in the membrane and induces Ca^2+^-dependent protein kinase C (PKC), which in the course of the induction of mitogen-activated protein kinases (MAPK) [50,51] leads to gondotropin synthesis (LH and FSH) and secretion [50,52,53,54]. The signaling found to be activated by GnRH agonists in the pituitary is not turned on in cancers of the ovary, although PLC, PKC and AC can be activated in these cells by pharmacologic stimulation. [40,55].

**Figure 1:**
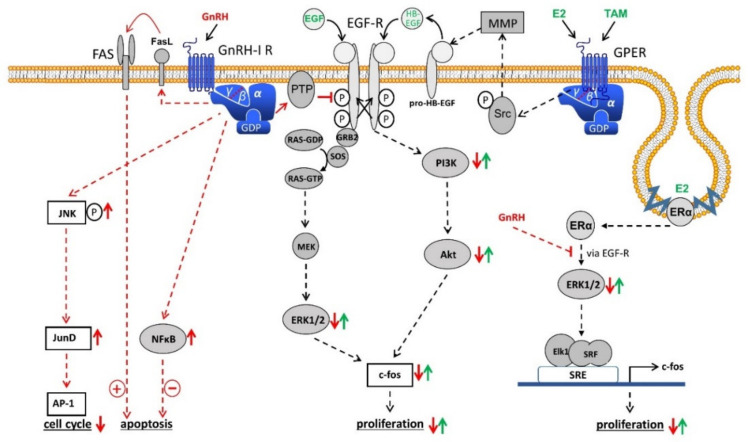
Schematic representation of GnRH-I receptor signaling in ovarian cancer cells. Binding of GnRH-I and GnRH-I analogs causes G-protein αi-mediated activation of a PTP, resulting in dephosphorylated EGF-R and inhibition of EGF-R signaling through ERK1/2 or PI3K/AKT pathway leading to reduction of proliferation. GnRH-I-induced activation of PTP also inhibits the signal transduction of GPER and membrane-associated ERα through transactivation of EGF-R. In addition, GnRH induces activation of NFκB to reduce and FAS-ligand to increase apoptosis. Finally, GnRH agonists activate the JNK/AP-1 pathway resulting in increased G0/1 phase of the cell cycle and decreased synthesis of DNA. Further details are described in the text. Abbreviations: Akt, protein kinase B (PKB); AP-1, activator protein-1; E2, estradiol; EGF, epidermal growth factor; EGF-R, EGF receptor; ERα, estrogen receptor α; ERK1/2, p44/42 mitogen-activated protein (MAP) kinase; FAS, Fas receptor; FasL, Fas ligand; GDP, guanosine diphosphate; GnRH, gonadotropin-releasing hormone; GPER, G-protein coupled estrogen receptor; GRB2, growth factor receptor-bound protein 2; GTP, guanosine triphosphate; HB-EGF, heparin-binding EGF-like growth factor; JNK, c-Jun N-terminal kinase; JunD, transcription factor JunD; MEK, mitogen-activated protein kinase kinase (MAP2K); MMP, matrix metalloproteinase; NFkB, nucleus factor kB; PI3K, phosphoinositide 3-kinase; PTP, protein tyrosine phosphatase; RAS, G-protein rat sarcoma; SOS, guanine nucleotide exchange factor “son of sevenless”; SRE, serum response element; Src, tyrosine kinase cellular sarcoma; SRF, serum response factor; TAM, tamoxifen.

In contrast to the pituitary gland, the GnRH receptor-mediated signal transduction in gynecological tumors takes place via coupling with G-protein αi instead of G-protein αq, which may result in variable receptor conformations and signaling complexes [55,56,57] (Figure 1). Since the gene sequence of GnRH receptors in human carcinomas of the ovary is the same as that in the pituitary gland, different receptor conformations may explain why GnRH receptors in ovarian cancer show different mechanisms compared with cells derived from the pituitary. After binding to G-protein αi, the activated GnRH receptor induces a phosphotyrosine phosphatase (PTP) and prevents the signaling of growth factor receptors, which leads to a reduction in proliferation of cancer cells [40,55,58,59,60,61]. Growth factor-driven mitogenic signal transduction is prevented which leads to downregulation of growth factor-mediated activation of mitogen-activated protein kinase (MAPK) [40], *c-fos* expression [62], and growth factor-induced proliferation [63]. These findings are in agreement with other research on GnRH analogs showing reduction of growth factor receptor expression [64,65,66] and/or growth factor-induced tyrosine kinase activity [40,58,59,61,65,67,68,69]. Induction of apoptosis does not appear to be involved in the down-regulation of cancer cell proliferation by GnRH agonists [20]. In contrast, Imai et al. have reported, that GnRH agonist leuprorelin stimulated intratumoral expression of apoptosis-inducing Fas ligand in GnRH receptor-positive tumor cells. At a concentration of 10 µM, leuprorelin induced up to a 90% reduction in cell number preceded by Fas ligand production [70,71,72,73]. However, GnRH agonist triptorelin did not induce apoptosis. On the contrary, by activating NFκB, triptorelin even seems to protect against apoptosis [74,75].

In human cells from ovarian cancer, GnRH agonists act not only on the mitogenic signaling of growth factor receptors. They also induce the activation of activator protein-1 (AP-1). In addition, GnRH agonists promote activation of JNK, which triggers AP-1 [76]. In ovarian cancer cells, GnRH agonists stimulate neither phospholipase C (PLC) nor protein kinase C (PKC) [40]. In addition, GnRH agonists inhibit the mitogen-activated protein kinase (MAPK, ERK) activity induced by growth factors [40]. Therefore, GnRH-induced JNK/AP-1 activation depends not on the AP-1 activators PKC or MAPK (ERK).

Since the JNK/c-jun signal transduction is activated by antiproliferative GnRH agonists and since JNK/c-jun is integrated into the reduction of cell growth in different systems, it seems plausible that the JNK/c-jun signal transduction participates in the inhibiting effects of GnRH agonists. GnRH agonists have also been shown to induce the binding of JunD to DNA, resulting in a reduction of cell growth, as evidenced by an increased G0/1 phase of the cell cycle and reduced synthesis of DNA [77].

Expression of the immediate-early response gene c-*fos* is induced by 17β-estradiol (E2) in estrogen receptor α (ERα)-positive ovarian cancer cell lines [78,79,80,81,82,83,84,85]. Via MAPK-dependent phosphorylation of Elk-1 the serum response element (SRE) is activated [86,87]. Since GnRH agonists inhibit EGF-induced cell proliferation and expression of c-*fos* through Ras/MAPK, it was interesting to know whether activation of SRE and expression of c-*fos* induced by E2 in ERα-positive cancer cells is also influenced by GnRH agonists and whether GnRH inhibits E2-driven cell proliferation [20]. Resting ERα-positive/ERβ-positive cancer cell lines were stimulated to proliferate by treatment with E2. Simultaneous treatment with GnRH agonists prevented this effect in a time- and dose-dependent manner [88]. ERα-negative/ERβ-positive cell lines were not affected by treatment with E2. Furthermore, E2 activates serum response element (SRE) and expression of c-fos in ERα-positive / ERβ-positive cell lines. This was inhibited by GnRH agonists [88]. GnRH agonists had no influence on the activation of estrogen response element (ERE) caused by E2. Transcriptional activation of SRE by ERα is due to activation of the mitogen-activated protein kinase (MAPK) pathway. GnRH inhibits this signal transduction, resulting in a decrease in E2-induced SRE activation followed by a decrease in E2-mediated c-*fos* expression. This is causative for a reduction in E2-induced cell growth [88].

Activation of PTP by GnRH also inhibits Src/MMP/HB-EGF signaling via the G-protein βγ subunit of G-protein coupled estrogen receptor 1 (GPER1), a membrane-bound estrogen receptor [89,90,91,92]. Due to the inhibition of GPER signaling, cancer cell proliferation by E2 in breast cancer cells without expression of ERα was inhibited [89,90,91]. In human breast cancer cells, GnRH analogs counteract EGF-dependent proliferation. GnRH analogs likely disrupt the change in growth regulation from estrogen dependency to EGF dependence that occurs after the acquisition of secondary resistance against 4OH-tamoxifen. This disruption of EGF receptor signal transduction re-sensitized the resistant cell lines for a therapeutical use of 4OH-Tamoxifen [93]. Especially in breast cancer, GnRH is not only involved in cancer cell proliferation. In metastatic breast cancer cells, GnRH inhibits cell invasiveness in vitro and metastasis in vivo [94,95,96,97,98]. In addition, it was shown that GnRH is involved in epithelial to mesenchymal transition (EMT) [98]. However, these effects of GnRH still have to be researched in ovarian cancer in the future.

### 3.3. Expression of GnRH-II and Its Receptor in Ovarian Cancer

**Figure 2:**
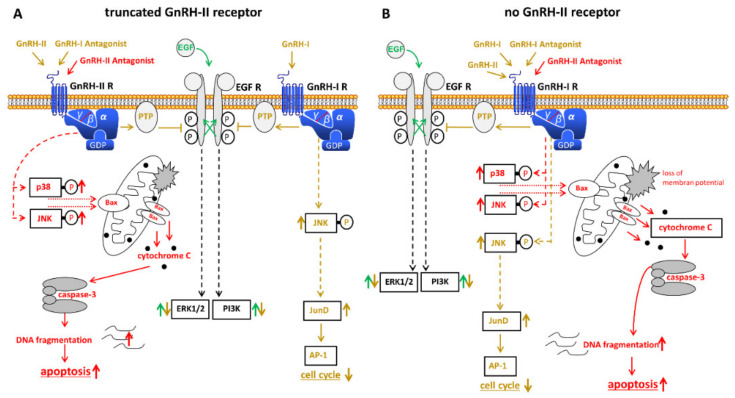
Schematic representation of GnRH-II signaling in ovarian cancer cells assuming that a truncated GnRH-II receptor (**A**) or no GnRH-II receptor (**B**) exists. (**A**) Binding of GnRH-II agonists, GnRH-I antagonists and GnRH-II antagonists to the truncated GnRH-II receptor cause PTP activation leading to dephopsphorylation of activated EGF-R. In addition, GnRH-II antagonists induce apoptosis through activation of p38, JNK and the intrinsic apoptotic pathway. GnRH-I agonists only couple to the GnRH-I receptor. Signal transduction mechanisms and further details are described in Figure 1 and the text. (**B**) Signal transduction of GnRH-I and GnRH-II and their agonistic as well as antagonistic analogs through the GnRH-I-R if no GnRH-II-R exists. Signaling and details are described in the text. Abbreviations: AP-1, activator protein-1; Bax, B-cell lymphoma 2 (Bcl-2)-associated X protein; EGF, epidermal growth factor; EGF-R, EGF receptor; ERK1/2, p44/42 mitogen-activated protein (MAP) kinase; GDP, guanosine diphosphate; GnRH, gonadotropin-releasing hormone; JNK, c-Jun N-terminal kinase; JunD, transcription factor JunD; P38, mitogen-activated protein kinase P38; PI3K, phosphoinositide 3-kinase; PTP, protein tyrosine phosphatase. Signal transduction pathway affected by GnRH-I agonists, GnRH-I antagonists, and GnRH-II agonists are marked brown. GnRH-II antagonist-induced signaling is marked red.

In addition to the well-known GnRH, many mammals express a second structural version of GnRH. The so-called GnRH-II is completely identical in its amino acid sequence from fish to mammal and differs from GnRH in three amino acids. A specific, functionally active receptor for GnRH-II has been found in many species. This also includes non-human primates [99,100,101,102]. Whether there is a GnRH-II receptor in humans is controversial [100,101,103,104,105,106,107,108]. There are, however, several indications for a functional GnRH-II receptor in humans [109] (Figure 2A). GnRH-II has inhibitory actions on human ovarian cancer cell proliferation that are significantly larger than those of the high active GnRH-I agonist triptorelin [104]. As well as GnRH-I agonists, GnRH-II agonists inhibit mitogenic signaling from growth factor receptors by activating a PTP. This leads to a down-regulation of the proliferation of cancer cells [55,62,110]. In contrast to GnRH-I and GnRH-II agonists and in addition GnRH-I antagonist cetrorelix, GnRH-II antagonists promote apoptosis in human ovarian cancer cells [111]. GnRH-II antagonist-induced apotosis is permitted via the intrinsic apoptotic pathway. This happens via the MAPKs p38- and JNK-induced activity of the pro-apoptotic protein Bax, followed by the loss of the mitochondrial membrane potential and subsequent cytochrome c release followed by caspase-3 activation [111,112]. Confirming these anti-tumor effects in nude mice it was shown that antagonistic analogs of GnRH-II significantly reduced the growth of human ovarian cancer xenografts in mice with no noticeable side effects [111].

SK-OV-3 cells are a cell line derived from ovarian cancer without GnRH-I receptor expression [23]. Here, the GnRH-I agonist triptorelin showed no effects on cell proliferation [23]. The GnRH-I antagonist Cetrorelix and GnRH-II, on the other hand, had a strong antiproliferative effect on this ovarian cancer cell line [113]. In addition, it was shown that in cell lines in which both, the GnRH-I agonist triptorelin and the GnRH-I antagonist cetrorelix act, only the effects of the former were abolished when the GnRH-I receptor was knocked down. The effects of cetrorelix and GnRH-II, however, were retained [113]. These results suggest that the proliferation reducing effects of the cetrorelix and of GnRH-II are not permitted via the GnRH-I receptor. These findings are in accord with results published by other researchers. Enomoto et al. [114] have shown that the human GnRH-II receptor is working and its splicing variant indicates the way of the cell response that follows GnRH-II stimulation. Expression of GnRH-II was detected in normal neoplastic cells of the ovarian surface epithelium and in cancers developed from these cells. Furthermore, they could show that GnRH-II had antiproliferative effects in immortalized cells from the ovarian surface epithelial [115].

However, at first glance, these findings appear to be in contrast to the fact that the human GnRH-II receptor gene has a stop codon in exon 2 [104,108]. The complete human GnRH-II receptor is known to be a 7-transmembrane receptor. Therefore, it was speculated that a working GnRH-II receptor might be a truncated 5-transmembrane domain receptor lacking transmembrane regions 1 and 2 [101]. This is in accord with data showing the expression of a GnRH-II receptor-like antigen in human ovarian cancer cells [109]. Photochemical reaction of ^125^I-labelled (4-azidobenzoyl)-N-hydroxysuccinimide-[D-Lys^6^]-GnRH-II with isolated cell membranes of human ovarian cancer cells provided a signal at about 43 kDa. A Western blot of the same gel showed that this signal could be a GnRH-II receptor-like antigen, which would indicate the existence of a shortened GnRH-II receptor [109]. In competition experiments, treatment with the GnRH-I agonist triptorelin led to a slight decrease in the binding of ^125^I-labelled (4-azidobenzoyl)-N-hydroxysuccinimide-[D-Lys^6^]-GnRH-II to its binding site. Treatment with the GnRH-I antagonist cetrorelix led to a significantly greater decrease, while the GnRH-II agonist [D-Lys^6^]-GnRH-II was the strongest competitor [109]. Indeed, these data suggest that the GnRH-II receptor-like antigen shown may be the specific binding site for GnRH-II sought.

On the other hand, it could be shown that GnRH-II antagonists as well bind to the GnRH-I receptor with affinities for the GnRH-I receptor that are comparable to those of the cetrorelix [112,116]. It was also shown that [D-Lys^6^]-GnRH-II has agonistic characteristics at the GnRH-I receptor, while GnRH-II antagonists clearly act as antagonists on the GnRH-I receptor [112]. This would mean that GnRH-I and -II antagonists, as clear antagonists, should not have any effects and are only block the GnRH-I receptor. Therefore, it was speculated that, besides the known autocrine GnRH-I system, a further autocrine system based on a GnRH-II could be present in human ovarian cancers.

Although there are the above mentioned indications for the presence of a working GnRH-II receptor in humans [104,109,113], other groups assumed that the human GnRH-II receptor is not functional. Kim et al. were able to show that the actions of GnRH-I and GnRH-II can be undone by transfecting siRNA in order to abolish expression of GnRH-I receptor [117]. These results suggest that the effects of GnRH-I and GnRH-II are permitted through the GnRH-I receptor (Figure 2B). These contradictions are a little confusing. Ultimately, it has not yet been possible to clarify whether there is a functional GnRH-II receptor in humans.

### 3.4. GnRH Analogs as a Possible Therapy for Ovarian Cancer

#### 3.4.1. Suppression of Pituitary Gonadotropin Secretion by GnRH Analogs

The most obvious strategy is the suppression of gonadotropin secretion by downregulation of the HPG axis using GnRH analogs. It was shown that in cancers of the ovary and ovarian sex-cord-stromal tumors, GnRH agonists might have antitumor effects permitted by inhibition of secretion of the gonadotrophins (reversible medical hypophysectomy) [26]. First, Parmar et al. reported a patient with advanced ovarian cancer who got a relapse after surgery, chemotherapy and radiation therapy and was then treated with triptorelin [118,119] (Table 1). Simultaneously with the suppression of gonadotropins, there was a significant shrinkage of the tumor mass over 12 months. Subsequently, several researchers showed that the reduction in LH and FSH levels achieved by administration of GnRH agonists in 10–50% of patients with advanced ovarian cancer who have relapsed after standard treatment, led to objective remissions or stable disease [118,119,120,121,122,123] (Table 1). Based on these promising data from in vitro experiments and clinical pilot studies, a prospective double-blind randomized clinical study was started in patients with advanced ovarian cancer who received surgical treatment and chemotherapy [124]. Unfortunately, this study showed no benefits of suppression of gonadotropins by treatment with GnRH agonist triptorelin in progression-free and overall survival [124] (Table 1). In a more recent phase II trial using tamoxifen and goserelin in a patient with recurrent epithelial ovarian cancer, no consistent correlation between suppression of LH and FSH and tumor response was found [125] (Table 1).

#### 3.4.2. Direct Treatment of Ovarian Cancer

In addition to the indirect use of GnRH analogs for suppression of LH and FSH, the expression of tumor cell GnRH receptors can be used to treat cancer cells directly. The easiest way to use the GnRH receptor as a therapeutic target is to use GnRH agonists or antagonists. Different GnRH agonists were developed and tested including clinical trials i.e., Triptorelin, Buserelin, and Goserelin. However, combinations of GnRH agonists with cytotoxic chemotherapy were not more efficacious than cytotoxic chemotherapy alone. This may be due to the fact that the doses were too low for a direct effect on the tumor cells.

Since GnRH agonists could not meet the expectations in the clinical settings, further trials were focused on the use of high-dose GnRH antagonists. GnRH antagonist Cetrorelix is able to safely and effectively inhibit the secretion of gonadotropins. Due to its suppression of LH and sex steroid hormones, Cetrorelix has been used to treat hormone-dependent cancers. Cetrorelix is also used to inhibit the premature LH surge in controlled ovarian hyperstimulation [136]. Cetrorelix has also been shown to have a direct anti-tumor effect in GnRH receptor-positive cancer cells. In addition, Cetrorelix had better anti-tumor efficacy than GnRH agonists in in vivo models of human cancers. Therefore, a phase II clinical trial with a high dose of GnRH-I antagonist Cetrorelix was performed in patients with ovarian or müllerian carcinoma refractory to platinum chemotherapy [137]. Eligible patients received 10 mg of cetrorelix subcutaneously every day. Three of 17 evaluable patients treated with Cetrorelix, obtained partial remission (18%) which lasted for 2 to 6 months. Six patients did undergo disease stabilization (35%) for up to 1 year [137]. In this very refractory patient group, these results are quite impressive when compared with chemotherapy under palliative situations. Only minimal toxicity was found, except for potential anaphylactoid reactions [137]. One patient had a grade 4 anaphylactoid reaction controlled by cortisol and cimetidine, two patients developed a grade 2 histamine reaction, one patient had a grade 2 arthralgia, and two patients had a 20% cholesterol increase. In addition, minor hot flushes, headaches, or local skin reactions at the site of injection were observed [137].

Despite successful testing of GnRH-II antagonists in vitro and in nude mice [103,111,112], this strategy has unfortunately not been continued so far. This may be due to the fact that there might be no functional GnRH-II receptor in humans [102].

Since peptide GnRH analogs have to be given by subcutaneous injection, small non-peptide GnRH analogs for oral administration were developed and successfully tested, even in clinical trials [138,139,140,141,142,143,144].

#### 3.4.3. GnRH Receptor Target Therapies

A third strategy was the use of GnRH receptors for a targeted therapy. Expression of GnRH receptors could only be detected in a few normal tissues such as organs from the reproductive tract and cells from the anterior pituitary. Other tissues and hematopoietic stem cells, on the other hand, do not express the GnRH receptor. In addition, ovaries, fallopian tubes and uterus are usually removed during surgery in ovarian cancer therapy [145]. The GnRH receptor is therefore very suitable as a target for therapies with improved anti-tumor effects and reduced side effects.

Cytotoxic GnRH agonists have been developed in which a cytotoxic substance is covalently linked to a GnRH agonist [146]. These so-called cytotoxic GnRH analogs bind specifically to the GnRH receptor with their peptide part and act as a chemotherapeutical drug after internalization of the receptor–ligand complex into the cell [146]. Therefore, these cytotoxic GnRH analogs only act in cells with GnRH receptors on the cell surface. As a result, they have significantly fewer side effects than non-conjugated cytotoxic substances [146]. The cytotoxic GnRH agonist zoptarelin doxorubicin (AEZS-108, AN-152) is such a fusion molecule. In zoptarelin doxorubicin, the cytotoxic part doxorubicin is covalently bound to the GnRH agonist [D-Lys^6^] GnRH. After GnRH receptor-mediated internalization of zoptarelin doxorubicin into the cell, the active part is split off and the now free doxorubicin accumulates selectively in the cell nucleus. The competitive inhibition of the uptake of zoptarelin doxorubicin by an excess of another GnRH agonist proves that zoptarelin doxorubicin enters the cell specifically via the GnRH receptor. In tumor cell lines that have no GnRH receptors, no intracellular zoptarelin doxorubicin could be found [147]. In addition, zoptarelin doxorubicin was significantly more effective than doxorubicin at the same dose at inhibiting cell growth in most ovarian cancer cell lines with GnRH receptor expression. Another advantage of this therapeutic strategy is the bypassing of the multidrug resistance protein-1 (MDR-1) resistance mechanism [148]. This mechanism of entry of zoptarelin doxorubicin may overcome the resistance conveyed by MDR-1, which is a major drawback of systemic therapies [148]. In addition, the targeting of zoptarelin doxorubicin to GnRH receptor-positive cancers makes this analog more effective and less toxic than doxorubicin [148].

In in vivo experiments with tumor-bearing mice, zoptarelin doxorubicin showed lower toxicity than free doxorubicin. In addition, zoptarelin doxorubicin showed better efficacy in inhibition of the growth of GnRH receptor-positive tumors [145,149]. This is probably due to the uptake of zoptarelin doxorubicin via the GnRH receptor and the resulting reduced resistance [148,150]. Zoptarelin doxorubicin has been used in women with GnRH receptor positive cancer in clinical dose-escalation and pharmacokinetic studies. The maximum tolerated dose without supportive drugs was 267 mg/m^2^. This dose was proposed for the therapeutic phase II studies [151] (Table 2). In 2014, the first data from a multicenter phase II study showed that zoptarelin doxorubicin is an effective and safe compound [152]. The activity and toxicity of zoptarelin doxorubicin were evaluated in 42 women with platinum-refractory or resistant GnRH receptor-positive ovarian cancer. The patients were treated with a zoptarelin doxorubicin dose of 267 mg/m^2^. This is equimolar to 76.8 mg/m^2^ of free doxorubicin. Six (14.3%) of these 42 patients had a partial response, 16 (38%) had stable disease, and 16 (38%) had progressive disease. Four patients could not be evaluated. The median time to progression was 12 weeks (95% confidence interval (CI): 8-20 weeks). Median overall survival was 53 weeks (95% CI: 39-73 weeks). The side effects were tolerable [152] (Table 2). In a phase III advanced endometrial cancer trial with unknown GnRH receptor status, median overall survival was not improved with zoptarelin doxorubicin [153]. Unfortunately, zoptarelin doxorubicin was discontinued and no phase III trial was conducted in ovarian cancer. Therefore, it remains unclear whether zoptarelin doxorubicin would have had an advantage in ovarian cancer. In addition, it is unclear, whether changes in study design would have shown better results. However, the success of most GnRH receptor-mediated therapy concepts led to a number of other promising approaches.

Based on tumor-specific signaling of GnRH receptor in gynecological cancers including ovarian cancer and specific distribution pattern of GnRH receptors, gene therapy by using a GnRH analogs as an inducer for transcription of a therapeutic gene was successfully developed and tested in vitro and in athymic mice bearing xenografts of ovarian cancer cells [154].

EP-100 a fusion peptide in which 18-amino-acid cationic α-helical lytic peptide (CLIP-71) is covalently linked to GnRH, was created to deliver lytic peptides to GnRH receptor-positive cancer cells [155] (Table 2). It was recently shown, that EP-100 in combination with PARP inhibitor olaparib is a promising strategy for the therapy of ovarian cancer [156].

Expression of the GnRH receptor can not only be used for target therapies. The selective expression of the GnRH receptor is also suitable to detect metastases that also express the GnRH receptor. Liu et al. have successfully developed a selective fluorescence probe to detect peritoneal metastases of ovarian cancer. For this purpose indocyanine green was conjugated to a GnRH antagonist [157].

## 4. Conclusions

GnRH is an important player in the control of the HPG axis. In addition to this classical hypophysiotropic function, GnRH is important as a regulator of cell proliferation and invasion in a number of human malignancies, including ovarian carcinoma. Furthermore, the selective expression of GnRH receptors makes it an ideal target for new effective therapeutic strategies with little side effects. First studies using GnRH analogs for direct tumor treatment were only moderately successful. Probably the used dosages were too low. Therefore, further studies focused on the use of high-dose GnRH analogs showing better efficacies. Following the discovery of GnRH-II, analogs were developed and successfully tested in vitro and in vivo. Since the existence of a functional GnRH-II receptor in humans is still controversial, this strategy has not been continued. The most promising strategy so far is the use of the GnRH receptor for target therapy. Cytotoxic GnRH agonist zoptarelin doxorubicin was shown to be an effective and safe compound. No phase III trial was conducted in ovarian cancer so far. Even if a single phase III study was unsuccessful, it still makes sense to pursue the concept further. In addition, the specific expression of GnRH receptors could be used diagnostically to detect metastases.

## Figures and Tables

**Table 1:** Clinical trials—Suppression of pituitary gonadotropin secretion through GnRH-analogs as treatment for epithelial ovarian cancer.

Reference	Pat.	Age	Diagnosis	Drug	Results
[118]	41	42–79	advanced ovarian cancer	Triptorelin depot	PR: 6 SD: 5
[120]	5	50–64	refractory ovarian cancer	Leuprolide acetate 1 mg/day	CR: 1 PR: 4
[121]	19 11 untreated	38–73	progressive ovarian cancer	Triptorelin 0.1 mg/day or 3.2 mg/month depot	SD: 12
[122]	18	21–68	progressive ovarian cancer	Leuprolide acetate 1.0 mg/day	PR: 4 SD: 2 NE: 5
[124]	69 66 placebo	<44–>75	stage III or IV epithelial ovarian cancer	Triptorelin 3.75 mg/month depot	No significant differences in progression free and overall survival
[125]	26	49–79	advanced ovarian cancer	Tamoxifen 20 mg/month Goserelin 3.6 mg/month	CR: 1 PR: 2 SD: 10
[126]	30	38–90	relapsed epithelial ovarian cancer	Goserelin 3.6 mg/month	PR: 2 SD: 5
[127]	25	median 65.5	advanced epithelial ovarian cancer	Leuprolide acetate	PR: 4 SD:15
[128]	20	average 60	progressive ovarian cancer	Triptorelin 3.75 mg/4 weeks	SD: 14
[129]	32		relapsed epithelial ovarian cancer	Leuprolide acetate 3.75 mg depot	PR: 4 SD: 5
[130]	14	47–77	advanced epithelial ovarian cancer	Triptorelin 3.2 mg/28 days	SD: 8
[131]	68	42–87	relapsed ovarian cancer, mostly refractory to platinum	Triptorelin 3.75 mg/on days 1, 8 and 28 followed by 4-weekly	SD: 11
[132]	32	32–77	platinum-refractory ovarian cancer	Leuprolide 3.75 mg/month	CR: 1 PR: 2 SD: 4
[133]	37	27–75	platinum- and paclitaxel-refractory ovarian cancer	Leuprolide acetate 3.75 mg/4 weeks	SD: 4
[134]	12	45–68	advanced ovarian cancer	Leuprolide acetate 3.75 mg on days 1, 8, 28 followed by 28-day intervals	PR: 1 SD: 3
[135]	23	44–72	refractory ovarian cancer	Goserelin 3.6 mg/month	PR: 4 SD: 7

CR, complete response; PR, partial response; SD, stable disease; NE, not evaluable.

**Table 2:** Clinical trials—GnRH receptor target therapy for treatment of epithelial ovarian cancer.

Reference	Pat.	Age	Diagnosis	Drug	Treatment	Results
[151]	4	55 +/− 11	epithelial cancers of ovary, endometrium, or breast	Zoptarelin-Doxorubicin	10, 20, 40, or 80 mg/m^2^	maximum tolerated dose: 267 mg/m^2^
	6	59 +/− 5			160 mg/m^2^	
	7	48 +/− 11			267 mg/m^2^	
[152]	42	49 +/− 10	epithelial ovarian, fallopian tube or primary peritoneal cancer	Zoptarelin-Doxorubicin	267 mg/m^2^	PR: 6 (14.3%) SD: 16 (38.1%) PD: 16 (38.1%) NE: 4 (9.5%)
[155]	28 (F) 9 (M)	59 (39–80) 61 (39–80)	ovarian, endometrial, breast and other cancers	EP-100	Initial cohorts: 1–7, 0.6–7.8 mg/m^2^, *n* = 21 Later cohorts:: 7–11, 7.8–40 mg/m^2^, *n* = 16	recommended phase II dose: 40 mg/m^2^

F, female; M, male; PR, partial response; SD, stable disease; PD, progressive disease; NE, not evaluable.

## Data Availability

Not applicable.
